# Polyacrylamide/poly(2-(dimethylamino) Ethyl Methacrylate) Interpenetrating Polymer Networks as Drug Delivery Systems for Diclofenac Sodium

**DOI:** 10.3390/gels8120780

**Published:** 2022-11-29

**Authors:** Kristina Grigorova, Bistra Kostova, Dilyana Georgieva, Anton Apostolov, Elena Vassileva

**Affiliations:** 1 Laboratory on Structure and Properties of Polymers, Faculty of Chemistry and Pharmacy, University of Sofia, 1, J. Bourchier blvd., 1164 Sofia, Bulgaria; 2 Department of Pharmaceutical Technology and Biopharmaceutics, Faculty of Pharmacy, Medical University of Sofia, 2, Dunav Str., 1000 Sofia, Bulgaria

**Keywords:** interpenetrating polymer networks, hydrogels, diclofenac sodium, drug delivery system

## Abstract

Nowadays, modern pharmaceutical investigations are directed toward the design and production of drug delivery systems for achieving prolonged and controlled drug delivery. In this respect, the use of interpenetrating polymer networks (IPNs) is an opportunity in the preparation of polymer drug delivery systems with desired characteristics. This paper describes the synthesis and characterization of novel poly(2-(dimethylamino) ethyl methacrylate) (PDMAEMA) and polyacrylamide (PAAm)-based IPNs with different compositions and their application as diclofenac sodium delivery systems. The prepared IPNs were shown to possess phase-separated structures at the nano level, as revealed by SEM and TM-DSC. The IPNs’ composition was shown to determine the swelling behavior of these novel materials, and the inclusion of the charged IPN component (PDMAEMA) has changed the water molecules type diffusion from Fickian to non-Fickian, as revealed by the swelling kinetics study. Loading efficiency of diclofenac sodium and diclofenac sodium content in the polymer network was evaluated, and in vitro drug release experiments were carried out in order to estimate the ability of the obtained IPNs to control the release of the water-soluble drug.

## 1. Introduction 

Diclofenac sodium is a non-steroidal anti-inflammatory drug (NSAID) widely used in the treatment of osteoarthritis, rheumatoid arthritis and ankylosing spondylitis [1]. Diclofenac is 100% absorbed after oral administration. However, oral administration is characterized by two main problems, namely some common side effects such as gastrointestinal disorders, peptic ulcer and gastrointestinal bleeding, as well as a short biological half-life of about 1–2 h, which requires multiple dosing to maintain a therapeutic level of the drug in the blood. There are different strategies to overcome these problems. The first strategy focuses on minimizing the side effects using various transdermal delivery systems, including lipid vesicles, solid lipid nanoparticles, transfersomes, microemulsions and hydrogel patches [2,3,4,5,6,7,8]. However, the majority of these systems are characterized by a significant burst release and heterogeneous drug distribution [9,10]. It should be noted that even though large doses have been applied, only a relatively small amount of diclofenac permeates through the skin [11]. The second strategy aims at both eliminating the need for multiple dosing and decreasing the occurrence of adverse effects, which can be achieved by the inclusion of diclofenac in sustained-release dosage forms [12,13].

A promising strategy for overcoming the above-mentioned problems is the utilization of IPNs. A few studies have been conducted for developing IPNs for diclofenac sodium delivery using different polymers; however, most of them are based on microparticles with IPN structure but not to bulk hydrogels [14,15,16,17,18,19].

To our best knowledge, polymeric carriers based on PDMAEMA and PAAm, including their IPNs, have been used so far as neither drug carriers nor, in particular, as diclofenac sodium carriers. These two polymers were chosen due to the fact that they have been reported to be non-toxic and biocompatible, and they have been widely used in recent years for the preparation of drug delivery systems [20,21,22,23,24,25,26]. It should be mentioned that PDMAEMA swells to a great extent in the stomach due to its pH-sensitive swelling behavior, which makes it inappropriate for oral drug delivery [27,28]. However, the addition of PAAm in the system will significantly reduce PDMAEMA swelling in acidic pH and will make it more appropriate as a carrier for the oral application of diclofenac sodium. Therefore, the aim of the present study is to prepare PDMAEMA and PAAm-based IPNs with varying compositions in order to achieve prolonged release of diclofenac sodium.

## 2. Results and Discussion

### 2.1. Equilibrium Swelling Degree in Water

**Figure 1:**
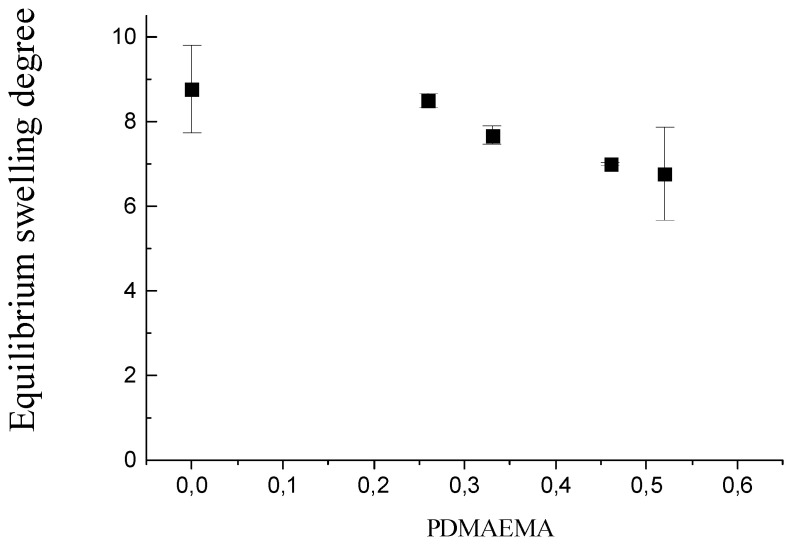
Dependence of equilibrium swelling degree in water of PDMAEMA/PAAm IPNs on their composition, φ_PDMAEMA_.

ESD of PDMAEMA/PAAm IPNs as a function of their composition (φ_PDMAEMA_) is presented in Figure 1. ESD of PDMAEMA/PAAm IPNs decreases as the content of PDMAEMA increases. This could be explained by the interlacing between PAAm and PDMAMEA chains which occurs in their IPNs. The number of entanglements between both networks increased with the increase of PDMAEMA content, which resulted in a higher crosslinking density of the IPNs as the entanglements play the role of physical network junctions. The PAAm network showed the highest ESD, which could be explained by the lack of the additional entanglements observed in the IPNs. Thus, by varying the IPN composition, the swelling ability of these networks could be controlled, and hence, the drug release profiles could be modulated.

### 2.2. Swelling Kinetics

**Figure 2:**
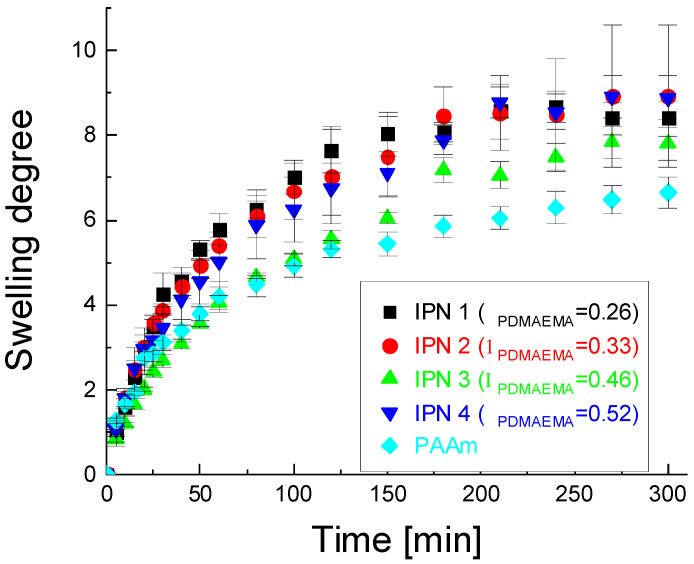
Swelling kinetics of the PDMAEMA/PAAm IPNs in water. For comparison, the PAAm single network swelling curve is also provided.

Although the single PAAm network had the highest ESD, it swelled very slowly during the first 6 h, as demonstrated by the swelling kinetics study (Figure 2). In contrast, all PDMAEMA/PAAm IPNs almost reached their ESD within these 300 min, i.e., they swelled much faster than the single PAAm network. This could be explained by the different rigidity of the polymer networks of the single PAAm network as compared to the IPN stricture, which was expected to result in different diffusion modes of water molecules. To study the swelling process in more details, the *M_t_*/*M_∝_* ratio was determined using Equation (3) as it could reveal the type of water transport in both polymer networks—the single PAAm network and the IPN. The time dependence of *M_t_*/*M_∝_*_,_ from which the exponent n and the coefficient k in Equation (3) could be determined, is presented in Figure S1 for IPN 4, (φ_PDMAEMA_ = 0.52) (see the Supplementary Information). The obtained values for the exponential factor n for all IPN compositions (averaged by three independent measurements) are presented in Table 1. The value n = 0.5 obtained for the single PAAm swelling in water indicates that water molecules obey Fickian diffusion when entering the single PAAm network. Fickian diffusion is observed when the rate of water molecules is slower than the rate of relaxation of the polymer chains, i.e., the polymer chains are very flexible and move faster as compared to water molecules. Therefore, the diffusion of water molecules into the PAAm single network is determined by their rate and is not influenced by the polymer chains, as the latter’s movements are much faster [29,30,31,32]. 

For all IPNs with different PDMAEMA/PAAm ratios, the value of n was higher than 0.5 but did not exceed 1. This indicated that the diffusion of water molecules in all IPNs was anomalous (non-Fickian), and the relaxation rate of IPNs polymer chains was comparable to the rate of water diffusion due to the entanglements of the polymer chains and, respectively, the increased density of IPNs compared to PAAm network (as already observed by the swelling experiments). These additional constraints reduce the mobility of the molecular segments of the IPN chains as compared to the PAAm chains in the single PAAm network and slow down their relaxation, making its rate comparable to the water diffusion rate. Thus, the diffusion of water in IPNs was a relaxation-controlled process, in contrast to the diffusion of water in the PAAm network, which was a diffusion-controlled process. It was expected that this result would also affect the drug release from the IPNs following the idea of the current study that the IPNs could control the drug release.

Using Equation (4), the diffusion coefficients of water molecules in the respective IPNs were also determined (Table 1). Their values confirmed the conclusion that water molecules diffused faster in IPNs as compared to the single PAAm network.

Our attempt to evaluate the crosslinking density increase of the IPNs due to the mutual interweaving and interlacing of both component networks by using two properties of the polymer networks sensitive to this parameter, namely elastic modulus and microhardness, fails to provide a clear estimation. The elastic modulus of the IPNs hydrogels at their ESD (Figure S2, Supplementary Information), as well as the microhardness of the dry networks (Figure S3, Supplementary Information), did not provide a clear dependence on the IPNs’ composition. Thus, the swelling degree appears more sensitive to the chain entanglement and interweaving as compared to the other two macroscopic properties of the polymer networks—their elastic modulus as well as microhardness. The observed different influence of the chain entanglements on the swelling ratio on the one hand and the mechanical properties (elastic modulus as well as microhardness) on the other hand could be explained by the fact that not all entanglements carry substantial load upon network deformation [33]. Thus, the entanglements could be divided into two groups: (i) load-bearing entanglements, which are between internal to the network chains (far from the chain end) and carry more stress as the deformation proceeds as well as (ii) load-non-bearing entanglements, which are the entanglements between the ends of the polymer chains. This assumption explains very well why the swelling studies are more sensitive and detect all entanglements existing in the IPN structure, while the mechanical properties detect only part of the entanglements, namely the ones that are load-bearing and directly participate in the mechanical properties’ evaluation. 

### 2.3. Scanning Electron Microscopy (SEM) 

**Figure 3:**
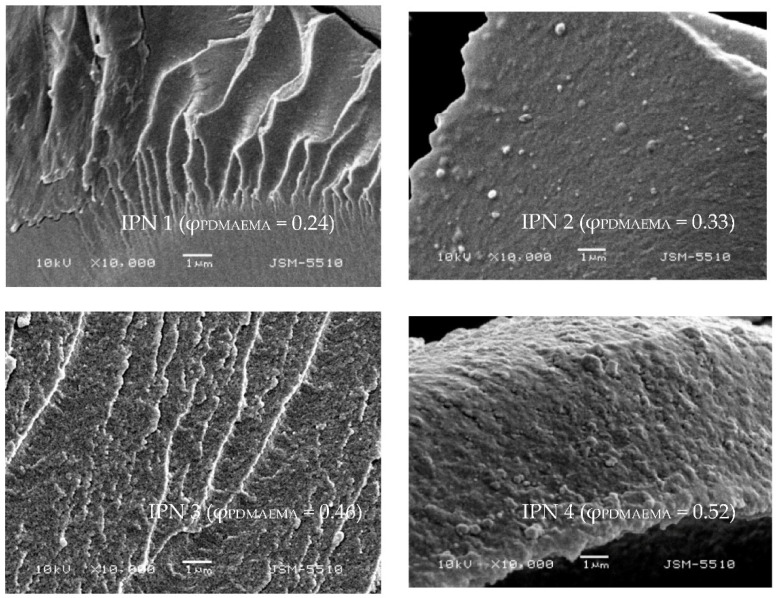
SEM micrographs of PDMAEMA/PAAm IPNs with different compositions (φ_PDMAEMA_) at magnification ×10,000.

The morphology of PDMAEMA/PAAm IPNs with different compositions (i.e., various φ_PDMAEMA_) was studied by SEM. At low magnifications (×1000), the broken surface of PDMAEMA/PAAm IPNs appeared smooth and uniform (Figure S4, Supplementary Information). At higher magnifications (×10,000 and above), however, the phase-separated structure of all PDMAEMA/PAAm IPNs was clearly seen (Figure 3). Figure 3 well presents the small (grain-like) PDMAEMA domains (white in the SEM pictures) dispersed into the PAAm matrix (gray in the SEM pictures). 

The PDMAEMA content increase resulted in an increase in the number and the size of the PDMAEMA domains, which was better illustrated at higher magnification (×30,000, Figure S5 and ×60,000, Figure S6, Supplementary Information). 

**Figure 4:**
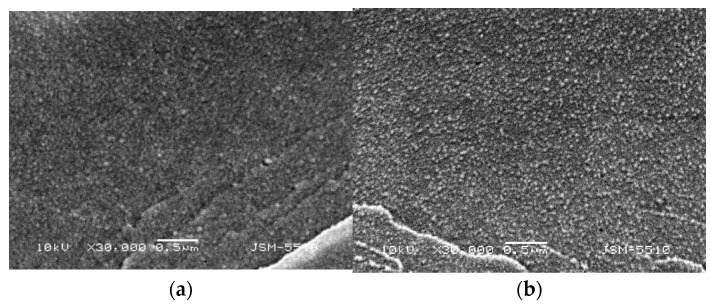
SEM micrograph of (**a**) IPN1 (φ_PDMAEMA_ = 0.26) and (**b**) IPN 4 (φ_PDMAEMA_ = 0.52) at magnification ×30,000.

SEM micrographs at higher magnifications were obtained in order to accurately estimate the size of PDMAEMA domains at different PDMAEMA content (Figure 4). For IPN 1 (φ_PDMAEMA_ = 0.26), the size of PDMAEMA domains was about 30 nm, while for IPN 4 (φ_PDMAEMA_ = 0.52), the size was above 200–400 nm (Figure 4). Moreover, the number of PDMAEMA inclusions in the PAAm matrix was higher in IPN4 as compared to IPN1 (Figure 4). This fact highlights the possibility of controlling the phase-separated structure of PDMAEMA/PAAm IPNs via the IPN composition, i.e., the phase-separated structure of IPN could be controlled. In Figure 4, it can also be seen that PDMAEMA domains were uniform in size and homogeneously distributed within the PAAm matrix. Moreover, the size of PDMAEMA domains was not bigger than 100 nm for smaller PDMAEMA content, e.g., IPN1 in Figure 4. Thus, the synthesized IPNs were, in fact, polymer/polymer nanocomposites-nanosized PDMAEMA that was finely dispersed into the PAAm matrix. Moreover, in IPN 4 (φ_PDMAEMA_ = 0.52), the PDMAEMA domains had size~200–400 nm, but also the domains merged with each other, forming a network-like structure. All these variations in the structure of IPNs were expected to define different drug diffusion and, respectively, different drug release profiles, which were, in fact, controlled simply by the IPNs’ composition.

### 2.4. Thermal Properties of PDMAEMA/PAAm IPNs as Well as of the Single PAAm Network

**Figure 5:**
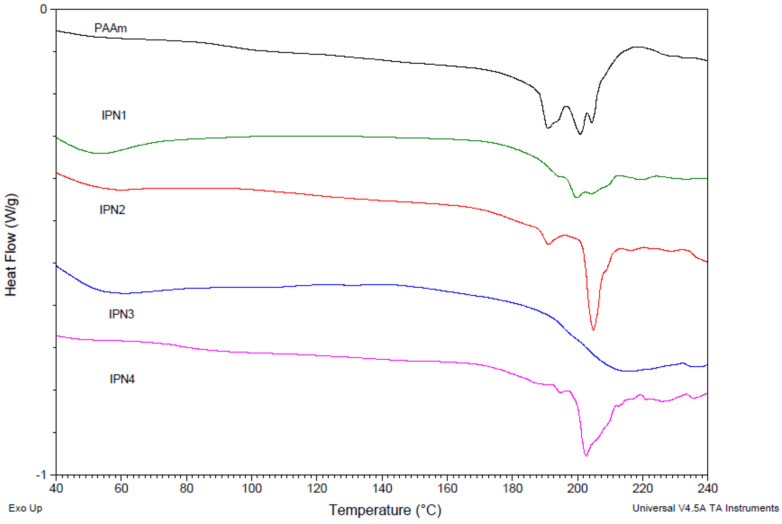
DSC thermograms of PDMAEMA/PAAm IPNs as well as of the single PAAm network.

The thermal properties of PDMAEMA/PAAm IPNs and PAAm single network were investigated using DSC (Figure 5) in order to confirm the observed by the SEM nano-sized dispersion of PDMAEMA into PAAm, i.e., IPNs’ nanostructure. An endothermic peak in the range of 190–210 °C was observed for the single PAAm network, which could be due either to crystal melting or thermal degradation. X-ray diffraction (Figure S7, Supplementary Information) confirmed that the PAAm single network did not have any crystalline fraction. Thus, the peak in Figure 5 is due to the PAAm thermal degradation. The results from the thermogravimetric analysis of PAAm (Figure S8, Supplementary Information) confirm this conclusion. 

In the thermograms of all IPNs, an endothermic high-temperature peak was observed, which could be attributed to the PAAm degradation as the X-ray analysis (Figure S7, Supplementary Information) confirmed the lack of crystallinity in all IPNs. Moreover, TGA and DTA analysis also indicated that IPNs undergo thermal degradation due to the PAAm component (Figures S9 and S10, Supplementary Information).

**Figure 6:**
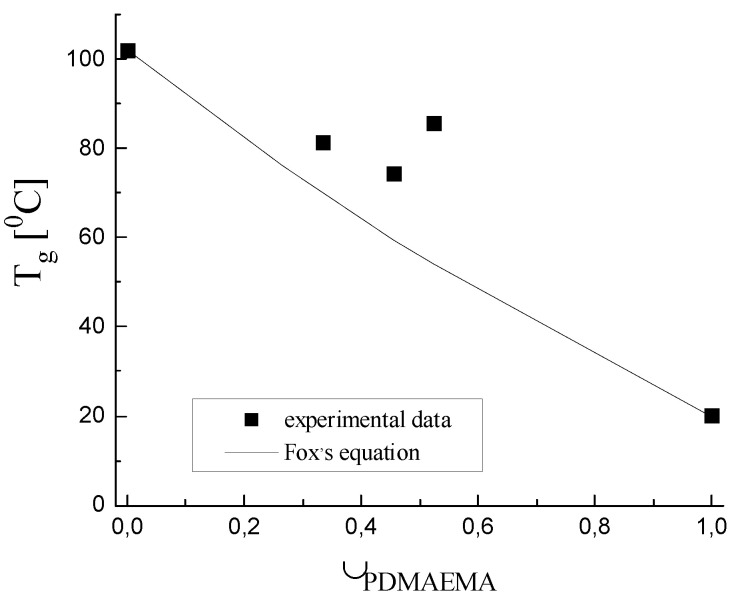
Relationship between the experimentally determined by DSC T_g_ of PDMAEMA/PAAm IPNs and their composition, φ_PDMAEMA_ (the black line is drawn following the Fox equation, Equation (S5) in Supplementary Information).

The glass transition temperature (T_g_) of PAAm is seen as a sigmoid change in the heat flow signal at 90–95 °C (Figure 5). According to the literature, the T_g_ of PDMAEMA is ~20 °C [34]. For the four PDMAEMA/PAAm IPNs, there is a well-defined glass transition, which was at a lower temperature as compared to the single PAAm network and at the same time above the T_g_ of PDMAEMA (Figure 6). This indicated that PAAm and PDMAEMA were mixed very finely, and although each of them formed a separated phase, as seen by SEM, the size of these phases was so small that they did not exhibit their own glass transition temperatures and only one glass transition temperature appeared in the DSC thermograms. The glass transition temperatures of the IPNs samples, obtained by the thermograms in Figure 5, are presented in Figure 6 as a function of the IPNs’ composition. As expected, all four IPNs had T_g_ lower than T_g_ of the PAAm network due to the fact that these materials are a combination of PAAm with a component with lower T_g_ (T_g_^PDMAEMA^ = 20 °C). 

For multicomponent polymer systems (blends, copolymers, etc.), the T_g_ dependence on their composition is usually described by the Fox equation (Equation (S5), Supplementary Information). However, as seen in Figure 6, the Fox equation did not describe well the experimental T_g_ dependence on the IPNs’ composition. One possible reason could be that the Fox equation did not take into account the possible interactions between the two components on the interfacial boundary in the IPNs (e.g., hydrogen bonds are formed between PAAm and PDMAEMA), which led to an increase in T_g_. Thus, the positive deviation in T_g_ above the Fox line (Figure 6), as well as the relatively wide glass transition intervals (Figure 6), could be explained by: (i) the strong interaction between the two IPNs’ components enhanced by the increased interfacial boundary between them within the phase separated IPN structure, as well as by (ii) the increase of IPNs’ density due to the increase of φ_PDMAEMA_. 

### 2.5. Diclofenac Sodium Content (%) in the Networks

The results obtained for diclofenac sodium loading in PDMAEMA/PAAm IPNs and PAAm single network are presented in Table 2. The results show that the IPNs’ composition did not significantly affect the amount of the loaded diclofenac sodium. Thus, it could be concluded that if different drug release profiles were observed for the IPNs with different compositions, they would be mostly due to the different structures of the IPNs, defined by their composition and, respectively, nanostructure and composition variation.

### 2.6. In Vitro Diclofenac Sodium Release Study

**Figure 7:**
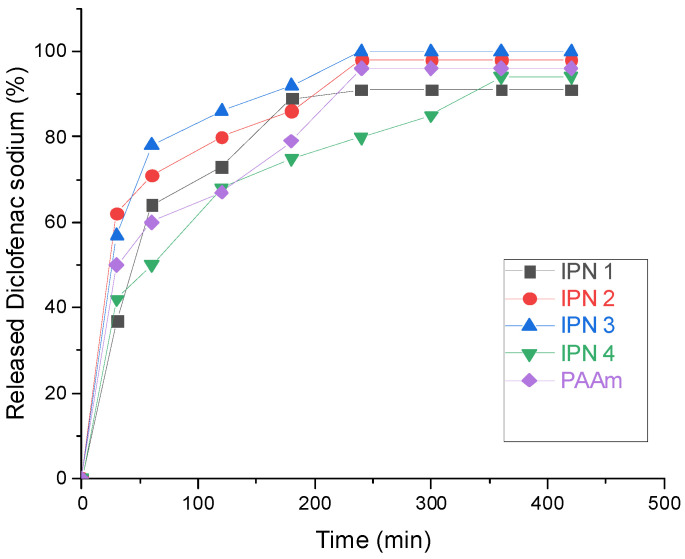
Drug release kinetics of diclofenac sodium at pH 6.8.

The results obtained from the in vitro diclofenac sodium release study are presented in Figure 7. It is clearly seen that the IPNs exhibit different drug release profiles depending on their composition. For the sake of comparison, the single PAAm network was also studied as a diclofenac sodium carrier. For the single PAAm network as well as for IPN 2 and IPN 3, a well-expressed “burst” effect was observed: within the first 30 min of the experiment, between 50 and 60% of the loaded drug was released (Figure 7). For IPN 1 and IPN 4, this effect was partially suppressed, and the amount of the released diclofenac sodium for the first 30 min was around 40%. The single PAAm network and all IPNs, except IPN 4, released diclofenac sodium very fast, and within the first 4 h of the experiment, more than 90% of the drug was already released. 

The best control over the diclofenac sodium release among the studied samples was achieved by IPN4, which demonstrated more steady and prolonged diclofenac sodium release after 6 h~90% of the loaded drug was released. This best performance among the other IPNs is due to several reasons, namely: (i) IPN4 has the highest PDMAEMA content, which component is positively charged and is expected to interact with the negatively charged diclofenac moiety, thus slowing down the drug release; (ii) the size of the PDMAEMA domains in the IPN4 structure is nanometric (~200–400 nm), but big enough to act as a reservoir for diclofenac sodium ensuring a constant release of the drug for a longer period of time and (iii) IPN 4 ensures polymer chain relaxation controlled drug diffusion which is expected to further slowdown the drug release. Thus, the combination of several parameters plays a crucial role in the prolonged diclofenac sodium release from this specific IPN composition. 

### 2.7. TM-DSC of Diclofenac Sodium Loaded PDMAEMA/PAAm IPNs 

**Figure 8:**
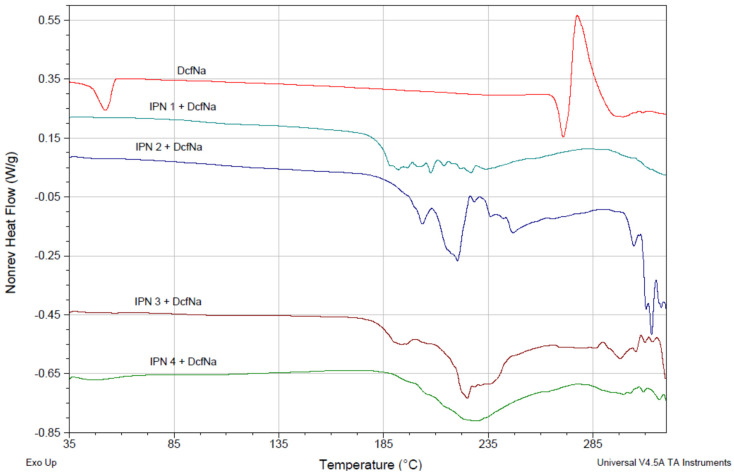
Non-reversing heat flows for diclofenac sodium-pure and loaded in PDMAEMA/PAAm IPNs with different compositions.

The interaction between diclofenac sodium and PDMAEMA/PAAm IPNs was studied by TM-DSC. Figure 8 presents the non-reversible heat flow for the IPNs, loaded with diclofenac sodium. The DSC thermogram of the pure diclofenac sodium showed a typical melting peak at 270 °C. When the drug was loaded into the polymer matrices, however, this peak did not appear in this temperature range which could be due to the amorphization of the loaded drug. This means that the polymer-drug interaction was strong enough to affect the drug crystallinity. 

**Figure 9:**
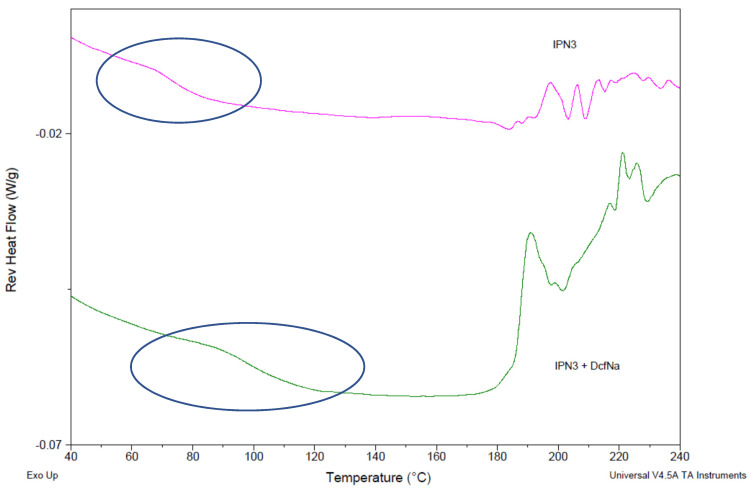
Reversing heat flow of IPN3 (φ_PDMAEMA_ = 0.46) without (red line) and with loaded diclofenac sodium (blue line).

This drug–polymer interaction also changed the properties of the IPNs. In Figure 9, the reversible heat flow for pure IPN3 (φ_PDMAEMA_ = 0.46) (purple line) and for diclofenac sodium-loaded IPN3 (green line) is presented. The drug loading resulted in an increase in the glass transition temperature of the IPN3 network, which was additional proof of the strong interaction between diclofenac sodium and PDMAEMA/PAAm IPNs.

## 3. Conclusions

In the present work, the potential of IPNs as delivery vehicles for prolonged diclofenac sodium release was demonstrated. PDMAEMA/PAAm IPNs were synthesized, and their characterization revealed their nano-structuring, observed by SEM as well as confirmed by TM-DSC. The IPNs’ composition affected the water molecules’ diffusion due to the changed relaxation rate of the polymer chains defined by the IPNs’ entanglements. Thus the PDMAEMA/PAAm IPNs composition was proved to be a tool to change the structure and hence the properties of these novel materials. The IPNs were successfully loaded with diclofenac sodium, and it appeared that the IPNs composition did not affect the amount of the loaded diclofenac sodium. The loading of diclofenac sodium into the IPNs led to drug amorphization, as well as to an increase in T_g_ of IPNs, as a result of the polymer–drug interaction. Based on the results obtained, the IPNs can be considered a promising approach for achieving prolonged drug release and, in particular, for the release of diclofenac sodium. This research could have a further impact on developing IPNs, based on other polymers as a tool to develop novel tailor-made systems for prolonged drug release where the IPNs components could be chosen according to the drug and the needed release profile. 

## 4. Materials and Methods

### 4.1. Materials 

From Acros, Belgium, 2-(dimethylamino) ethyl methacrylate (99%, DMAEMA) was obtained. Acrylamide (AAm) was obtained from Fluka, Germany, N, N’-methylene-bis (acrylamide) (MBAA) and potassium persulfate K_2_S_2_O_8_ (KPS) were obtained from Sigma-Aldrich, Germany. All reagents were used as received.

### 4.2. Methods

#### 4.2.1. Preparation of PDMAEMA/PAAm IPN

The sequential method for the IPN preparation was used where first the PAAm network was prepared, and then, the PDMAEMA network was formed in situ, using crosslinking polymerization. To this purpose, 50 mL 1 M aqueous solution of the monomer AAm was prepared by dissolving 3.55 g AAm in water using a volumetric flask of 50 mL. Then, 0.0135 g KPS (initiator) and 0.2929 g MBAA as crosslinking agents were added to this solution. In this way, their ratio to the monomer was respectively 0.1 mol% for KPS and 4 mol% for MBAA. The obtained solution was divided into ten equal parts, each one with a volume of 5 mL, and each 5 mL sample was placed in a closed container, and crosslinking polymerization was performed for 6 h at 60 °C. As a result, 10 samples of PAAm single polymer network were obtained, which were then thoroughly washed with distilled water for one week to remove the unreacted monomer, initiator and crosslinking agent. Two of the obtained networks were left as single PAAm networks in order to compare their properties with the further synthesized IPNs.

The second step was the in situ polymerization of the second network of PDMAEMA. To this purpose, 10 mL aqueous solutions of DMAEMA with different concentrations (0.3 M, 0.5 M, 0.7 M, 1 M) were prepared, also adding initiator (KPS) and crosslinking agent (MBAA) with ratio to the DMAEMA monomer in each solution respectively 0.1 mol% for KPS and 4 mol% for MBAA. In each of these DMAEMA solutions, one PAAm single network was immersed for 24 h. After 24 h swelling of PAAm networks in DMAEMA solutions, the resulting hydrogels were transferred into closed containers and subjected to crosslinking polymerization of DMAEMA at 60 °C for 6 h in order to obtain the corresponding IPNs. The volume of the residual DMAEMA solution not absorbed by the PAAm network after 24 h of swelling was measured. The difference between the initial volume of DMAEMA solution (10 mL) and the amount of unabsorbed DMAEMA solution after the end of the swelling was used to determine the molar part of PDMAEMA in the obtained IPNs (φ_PDMAEMA_ in Table 3). The aim was to obtain IPNs with different molar ratios of both constituent networks (PAAm and PDMAEMA) (Table 3). The obtained IPNs with different compositions (PAAm/PDMAEMA ratio) were then thoroughly washed with distilled water for one week to remove the unreacted monomer, initiator and crosslinking agent from the PDMAEMA polymerization.

Four IPN films with different compositions were synthesized, and good reproducibility in their composition was registered (Table 3).

#### 4.2.2. Swelling Kinetics in Water

The swelling kinetics of the IPNs and the single PAAm network was studied in distilled water. To this purpose, dry discs with a thickness of about 1 mm and a diameter of about 3 mm were cut from the IPNs as well as from the single PAAm network. Each disc was weighed and placed in 100 mL distilled water at 25 °C, measuring the weight change of the disc for 300 min. At defined time periods, the sample was removed from the water, whipped with fiber-free paper to remove the excess water and weighed. The swelling degree (*SD*) was calculated using the equation:

(1) $$SD=\frac{m_{swollen}-m_{dry}}{m_{dry}}$$

The *SD* of three disks from each IPN was determined, and the obtained values were averaged.

#### 4.2.3. Equilibrium Swelling Degree in Water

The equilibrium swelling degree (ESD) in water of each IPN was determined using 5 disks from each IPN composition. Their weight in dry state (*m_dry_*) was measured, and then each piece was placed in distilled water until it reached its equilibrium swelling degree, i.e., until the weight did not change furthermore within the experimental error (it took approximately 1 week). The ESD was determined by using the equation:

(2) $$\mathrm{ESD}=\frac{m_{swollen\;}^{e}-m_{dry}}{m_{dry}}$$

#### 4.2.4. Determination of the Diffusion Coefficient (D) of Water in the IPNs

The weight of water in the swollen disk from a given IPN (*M_t_*) at the moment t of the swelling kinetics and the mass of water in the same disk at its equilibrium swelling state (*M_∝_*) were determined using the data obtained from the swelling kinetics and the ESD of IPN and PAAm single network. The time dependence of the *M_t_/M_∝_* ratio was defined as follows:

(3) $$\frac{M_{t}}{M_{\propto }}=kt^{n}$$

where *k* is a constant characteristic of the polymer network, and *n* is the diffusion exponent, which is an indicator of the type of transport mechanism of water molecules in the polymer network. The diffusion coefficient of water molecules, *D*, was determined by using the equation:

(4) $$D={\left(\frac{k}{4\pi l^{2}}\right)}^{n}$$

where *π* is the Ludolph number (*π* = 3.14), and l is the sample thickness in meters.

#### 4.2.5. Scanning Electron Microscopy (SEM) 

The morphology of fractured surfaces of IPNs with various compositions was examined by scanning electron microscope JSM-5510, JEOL, Tokyo, Japan, operating at 10 kV. The samples were prepared for imaging by coating them with gold for 30 s using a sputter-coater JSC 1200, JEOL, Japan, under argon atmosphere.

#### 4.2.6. Temperature-Modulated Differential Scanning Calorimetry (TM-DSC)

DSC Q200 (TA Instruments, New Castle, DE, USA) was used to determine the thermal properties of dry IPNs and single PAAm network. Samples with a weight of about 5–8 mg were placed in T-zero pans, and the pans were tightly closed by a lid. An empty sealed T-zero pan was used as a reference sample. The heating chamber was purged continuously with N_2_ at a flow rate of 50 mL/min. The experiments were performed using TM-DSC heating mode with an amplitude of 1.0 °C/min for every 60 s; the heating rate was 2.0 °C/min in the temperature range of 25–250 °C.

#### 4.2.7. Loading Efficiency (LE)

Diclofenac loading into the *IPN*s was performed by immersing a piece of *IPN* with defined composition into 5 mL aqueous solution of diclofenac sodium (30 mg/mL) for 24 h. The LE (%) for each *IPN* was calculated using the weight of the dry samples as well as their weight after swelling for 24 h. 

The loading efficiency (%) of diclofenac sodium in *IPN*s and PAAm network was calculated by the following equation:

(5) $$Loading\;efficiency\;\left(\%\right)=\frac{m{\;}_{\left(IPN+DcfNa\right)}-m_{IPN}}{m_{\left(DcfNa\;in\;solution\right)}}\times 100$$

Diclofenac sodium content (%) was calculated using the equation:

(6) $$DcfNa\;content\;\left(\%\right)=\frac{m{\;}_{\left(IPN+DcfNa\right)}-m_{IPN}}{m{\;}_{\left(IPN+DcfNa\right)\;}}\times 100.$$

#### 4.2.8. In Vitro Drug Release Study

The drug release profiles were obtained by using an Erweka DT-GR apparatus (Erweka, Langen, Hessen, Germany). The paddle method was chosen by applying the following experimental parameters: 50 rpm, 37 ± 0.5 °C, 900 mL dissolution medium (pH 6.8). Filtered 5 mL samples were taken at regular time intervals to determine the released amount of diclofenac sodium. After each sampling, the volume was restored with 5 mL of dissolution medium. The amount of the drug released was determined spectrophotometrically at 276 ± 2 nm (Hewlett–Packard 8452 A diode Array spectrophotometer, NJ, USA), averaging it from 6 measurements.

## Figures and Tables

**Table 1:** n and D values for PDMAEMA/PAAm IPNs and PAAm network.

*φ*_PDMAEMA_	n	D [m^2^/s]
0	0.50 ± 0.03	1.34 × 10^−9^
0.26	0.78 ± 0.18	7.05 × 10^−9^
0.33	0.66 ± 0.05	5.30 × 10^−9^
0.46	0.644 ± 0.007	7.10 × 10^−9^
0.52	0.61 ± 0.03	9.19 × 10^−9^
1	-	-

**Table 2:** Diclofenac sodium loading in PDMAEMA/PAAm IPNs and PAAm single network.

Sample	Loading Efficiency (%) of Diclofenac Sodium in the Polymer Networks	Diclofenac Sodium Content (%) in the Polymer Networks
IPN 1	41%	17%
IPN 2	37%	16%
IPN 3	32%	14%
IPN 4	35%	15%
PAAm	38%	16%

**Table 3:** Composition of the synthesized PDMAEMA/PAAm IPNs expressed by the molar part of PDMAEMA in the obtained IPNs (φ_PDMAEMA_).

IPNs Designation	PAAm [M]	PDMAEMA [M]	φ_PDMAEMA_
PAAm	1	-	0
IPN 1	1	0.3	0.26
IPN 2	1	0.5	0.33
IPN 3	1	0.7	0.46
IPN 4	1	1	0.52
PDMAEMA	0	1	1

## Data Availability

Not applicable.

## Supplementary Materials

The following supporting information can be downloaded at: https://www.mdpi.com/article/10.3390/gels8120780/s1, Figure S1. Time dependence of *M_t_*/*M_∝_* for IPN 4 swelling in water. Figure S2. Dependence of the elastic modulus of PAAm single network and of PDMAEMA/PAAm on the IPNs’ composition, i.e., φPDMAEMA. Figure S3. Dependence of the microhardness of dry PDMAEMA/PAAm IPNs on φPDMAEMA. PAAm and PDMAEMA single networks microhardness values are provided for comparison. Figure S4. Micrographs of PDMAEMA/PAAm IPNs with different composition (φPDMAEMA) at magnification ×1000. Figure S5. Micrographs of PDMAEMA/PAAm IPNs with different composition (φPDMAEMA) at magnification ×30,000. Figure S6. Micrographs of PDMAEMA/PAAm IPNs with different composition (φPDMAEMA) at magnification ×60,000. Figure S7. X-ray diagrams of the four PDMAEMA/PAAm IPNs, as well as of the single PAAm network. Figure S8. TGA (green line) and DTA (blue line) of PAAm single network. Figure S9. TGA of the four PAAm/PDMAEMA IPNs. The TGA curves for the single PAAm and PDMAEMA networks are also provided. Figure S10. TGA (green line) and DTA (blue line) of IPN 4 (φPDMAEMA = 0.52).
