# Supplementary material for: Polyacrylamide/poly(2-(dimethylamino) Ethyl Methacrylate) Interpenetrating Polymer Networks as Drug Delivery Systems for Diclofenac Sodium
Source: Gels. 2022 Nov 29;8(12):780. doi: 10.3390/gels8120780 (PMC9778211; doi:10.3390/gels8120780)
Supplement: Supplementary file 1 [file gels-08-00780-s001.zip › gels-1963878-supplementary.pdf]

Supplementary Information

Swelling kinetics study

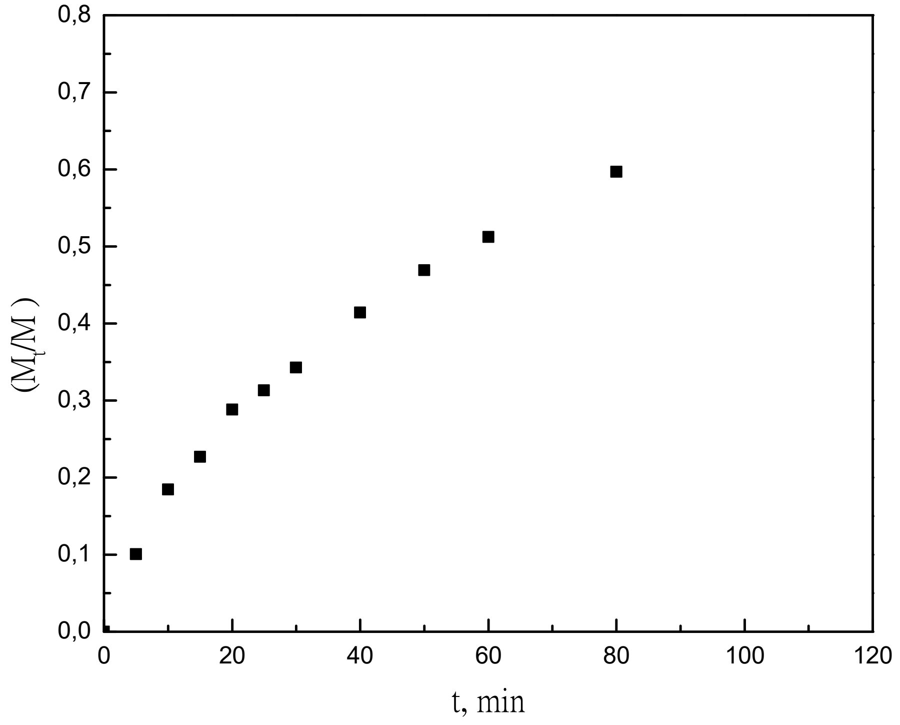

Figure S1. Time dependence of M_t/M_∞ for IPN 4 swelling in water.

Elastic moduli of PAAm and PDMAEMA/PAAm IPNs at their ESD

The elastic modulus of IPNs at their equilibrium swelling degree was determined according to the Hertz contact theory[1],[2]. For this purpose, a metal ball with weight W_{ball} and radius R_{ball} was placed on the surface of the respective hydrogel (Scheme S1). The ball sinks into the hydrogel at a depth D_{gel}. Using a cathetometer, the height of the ball above the surface of the hydrogel was measured 10 times. Based on the diameter of the ball, the value of D_{gel} was determined. Using D_{gel}, the radius of the contact surface between the gel and the ball $R_{contact}$ was calculated:

$$R_{contact} = (2R_{ball}.D_{gel} - D_{gel}^2)^{1/2} \qquad \text{(S1)}$$

To calculate the elastic modulus E_{gel} [kPa], the following relationship between E_{gel} and $R_{contact}$ was used:

$$\mathrm{E}\,gel = \left(\frac{9}{16}\right).\frac{R_{ball}W_{ball}g}{R_{contact}^3} \qquad \text{(S2)}$$

where W_{ball} and R_{ball} are respectively the weight and the radius of the ball, while g is the gravitational constant ($g = 9.8$ m/s^2). We used two balls with different weights and radii, namely ball 1 (radius = 5.5 mm, weight = 4.0704 g) and ball 2 (diameter = 7.5 mm and weight = 13.8185 g). Each of the E_{gel} values was obtained as an average of 10 independent measurements. The results are presented in Figure S2. The PDMAEMA content in the IPNs did not have influence on E_{gel}.

Our aim was to confirm that the physical network density (interchain entanglements in the IPNs) increased, using the parameter E_{gel} which is known to be sensitive towards the crosslinking density. Generally, the elastic modulus is known to increase with increasing the crosslinking density of the polymer networks. Since the equilibrium degree of swelling (Figure 2) showed increased IPNs density with increasing the PDMAEMA content, it was expected that the elastic modulus of the hydrogels would also increase with φPDMAEMA increase. However, according to the data presented in Figure S2, the IPNs composition, i.e. the increase in the PDMAEMA content, did not influence the IPNs' elastic moduli.

[1] H. Tamagawa, F. Nogata, K. Yagasaki "An interpretation of amphoteric gel hardness variation through potential and hardness measurement" *J. Coll. Int. Sci.* 275, 107 (2004)

[2] G.V.S.Henderson, D. O. Campbell, V. Kuzmicz, L. H. Sperling "Gelatin As A Physically Crosslinked Elastomer" *J. Chem. Edu.*, 62, 269 (1985)

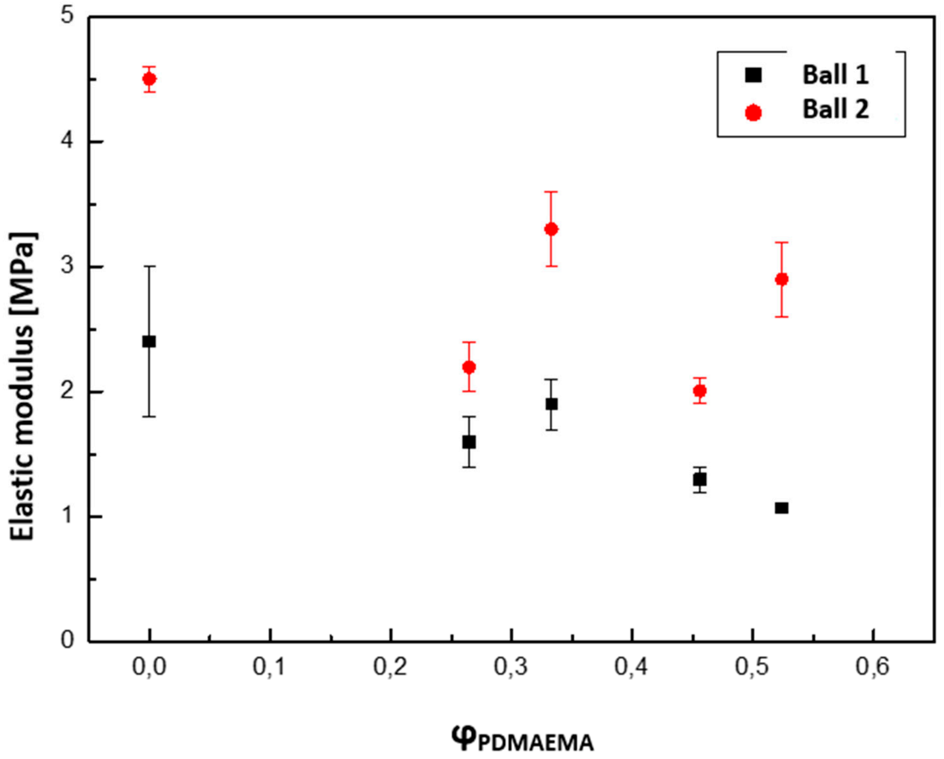

Figure S2. Dependence of the elastic modulus of PAAm single network and of PDMAEMA/ PAAm on the IPNs' composition, i.e. $\varphi_{PDMAEMA}$.

Vickers microhardness

Laika apparatus (Germany) was used to measure the microhardness of the dry IPNs and the single PAAm network. Three different forces, F [N], were used for indentation of the samples surface and for each force, the diagonals of the obtained indentation mark were measured 5 times. In this way, 10 values for each diagonal at a defined F value were obtained. The Vickers microhardness (HV) was calculated using the equation:

$$\mathrm{HV} = 1.854\frac{\mathrm{F}}{\mathrm{d}^2} \qquad \text{(S3)}$$

where F is the force [N], d [mm] is the mean value of the indentation mark diagonals.

The Vickers microhardness of the four IPNs and the single PAAm network is presented in Figure S3. There, the straight line, drawn according to the additivity law (eq. (4)), is also presented:

$$MH_{IPN} = MH_{PDMAEMA} \cdot \varphi_{PDMAMEMA} + MH_{PAAm} \cdot \varphi_{PAAM} \qquad \text{(S4)}$$

The results obtained for the microhardness of all IPNs did not show dependence on their composition, i.e. on the content of PDMAEMA. At values of φPDMAEMA below 0.5, IPNs showed microhardness lower than the predicted by the additivity law values. This could be related to the plasticizing effect of the water, absorbed from the air, in the samples.

The microhardness of PDMAEMA is reported to be 28 MPa.[3] PDMAEMA films were very thin and rubbery as the PDMAEMA glass transition temperature is ~ 20 ºC. PAAm network has high microhardness of ~ 470 MPa, which is explained by its high crosslinking degree - the crosslinking agent (MBAA) concentration is 4 mol% as well as partially by its elasticity – the latter defines a significant elastic recovery of the material after removing the indenter which results into a smaller indentation mark. The microhardness of PDMAEMA/PAAm IPNs was about twice lower than that determined for PAAm single network. Thus, the microhardness values of the IPNs could not be directly related to their crosslinking density as it was initially planned.

[3] Terzaki K, Kissamitaki M, Skarmoutsou A, Fotakis C, Charitidis CA, Farsari M, Vamvakaki M, Chatzinikolaidou M (2013). Pre-osteoblastic cell response on three-dimensional, organic-inorganic hybrid material scaffolds for bone tissue engineering. J Biomed Mater Res Part A 101:2283-2294.

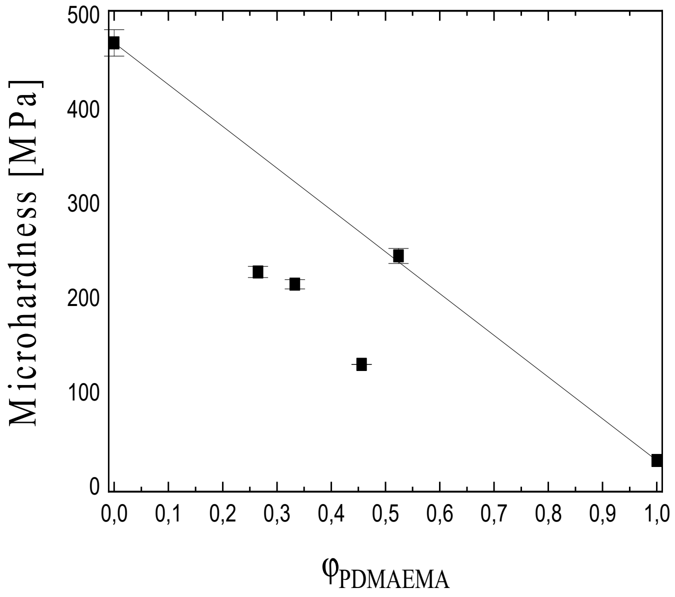

Figure S3. Dependence of the microhardness of dry PDMAEMA/PAAm IPNs on $\varphi_{PDMAEMA}$. PAAm and PDMAEMA single networks microhardness values are provided for comparison.

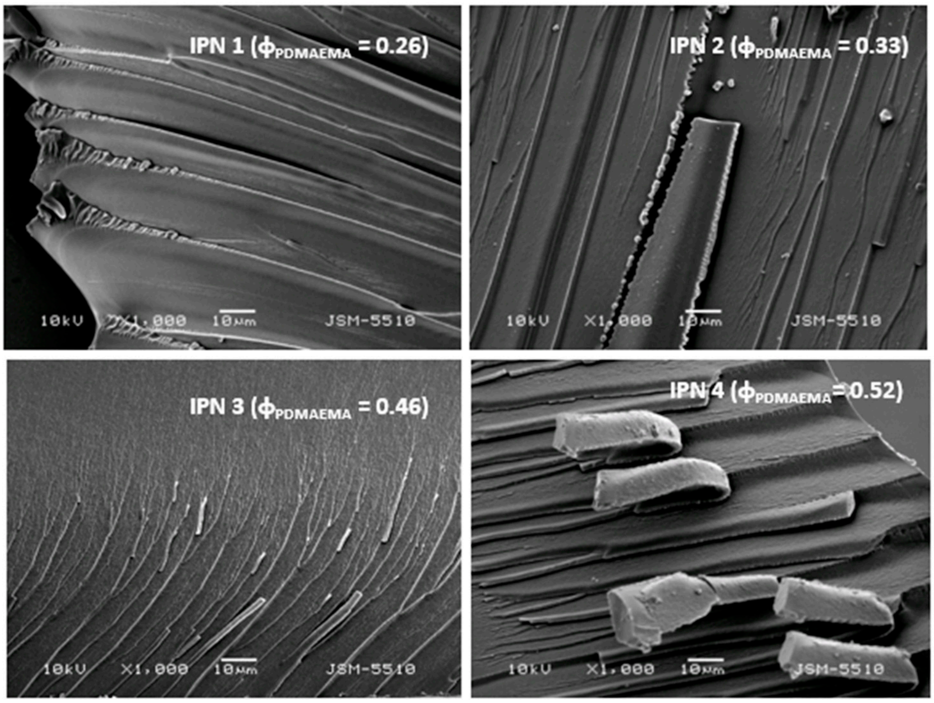

Figure S4. Micrographs of PDMAEMA/PAAm IPNs with different composition ($\varphi_{PDMAEMA}$) at magnification x1000.

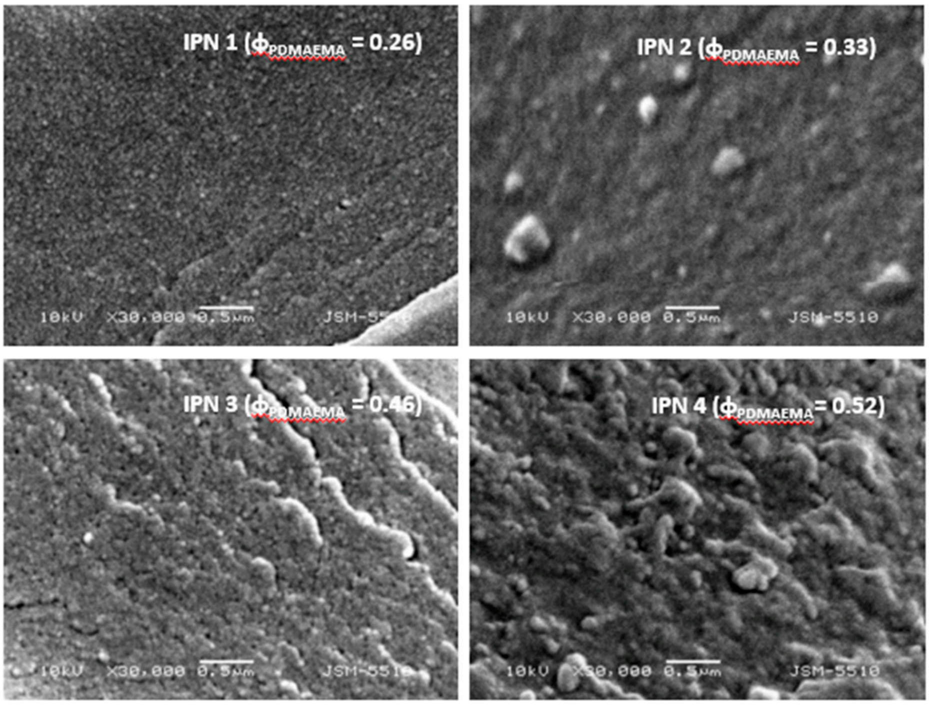

Figure S5. Micrographs of PDMAEMA/PAAm IPNs with different composition ($\varphi_{PDMAEMA}$) at magnification x30 000.

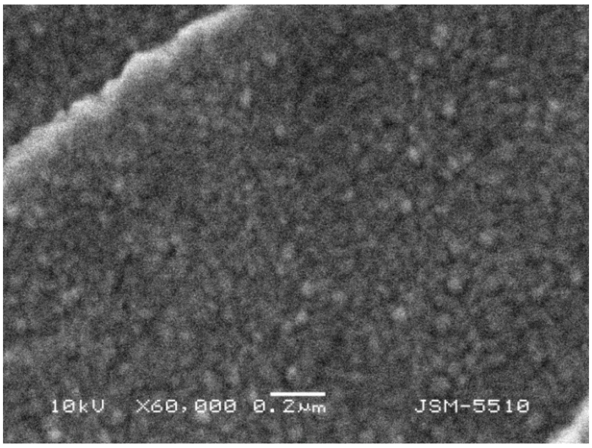

IPN 1 ($\phi_{PDMAEMA}$ = 0.26)

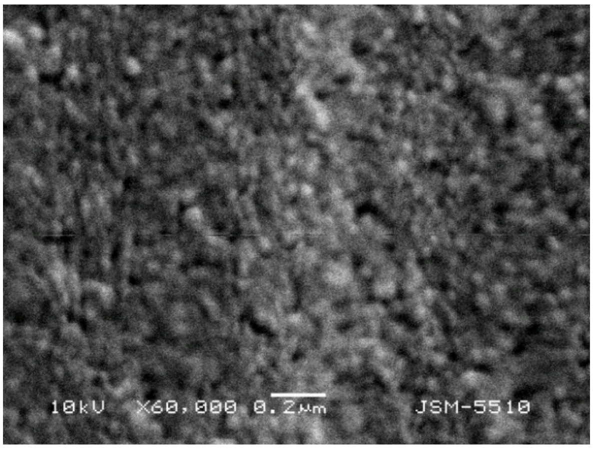

IPN 3 ($\phi_{PDMAEMA}$ = 0.46)

Figure S6. Micrographs of PDMAEMA/PAAm IPNs with different composition ($\varphi_{PDMAEMA}$) at magnification x60 000.

X-ray diffraction analysis (XRD)

A Siemens D500 diffractometer, Germany, with secondary monochromator and Cu-Ka radiation was used to obtain the diffractograms over 2θ range of 10–80° with a step of 0.03° and count time of 10 s.

Figure S6 shows the X-ray difractograms of all PDMAEMA/PAAm IPNs, as well as of the single PAAm network.

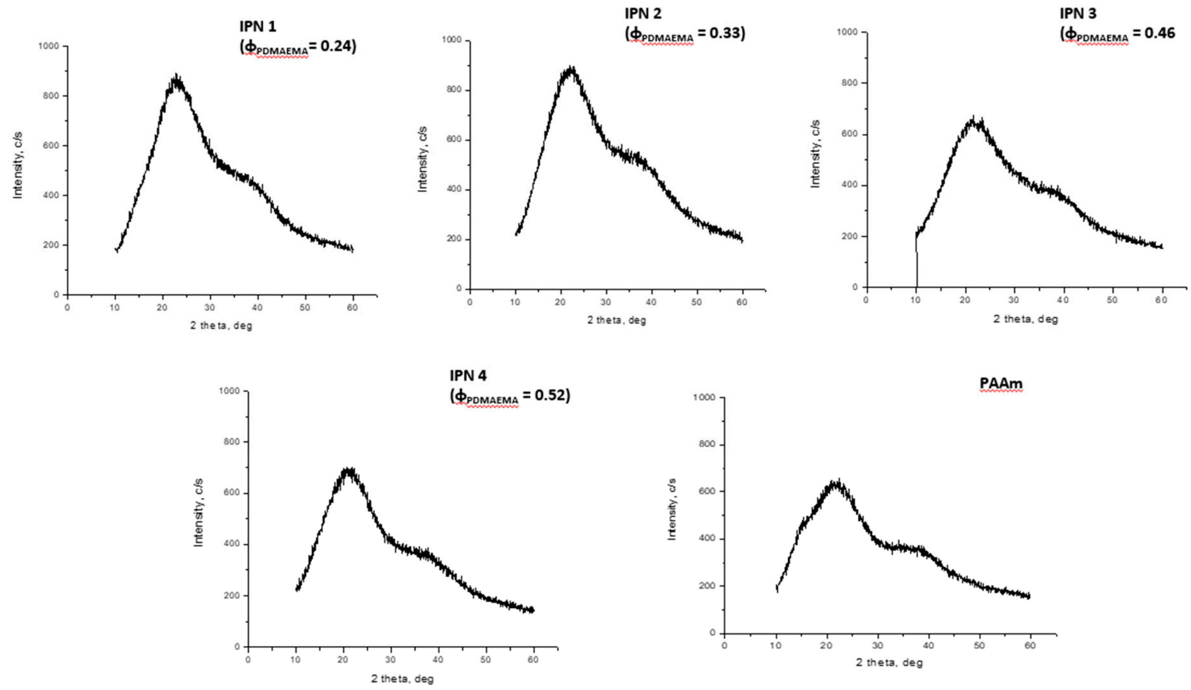

Figure S7. X-ray diagrams of the four PDMAEMA/PAAm IPNs, as well as of the single PAAm network.

The X-ray diffractograms of the four IPNs show that they are amorphous. This confirms that the high-temperature peak observed in Figure 5 is not due to melting but to decomposition of PAAm.

Thermogravimetric analysis (TGA)

Setaram, Labsys Evo 1600 differential thermal analyser was used to study the weight loss of the IPN samples. For this purpose, the samples were heated up to 300ºC in N_2 flow and the change in their weight was measured to evaluate their stability and degradation.

In Figure S8 are presented the curves obtained in a nitrogen atmosphere for PAAm single network by thermogravimetric analysis (TGA) and differential thermal analysis (DTA). PAAm single network shows a typical thermal degradation behavior at 220 °C (Figure S8). The observed endothermic peak at 224 °C in DTA curve was accompanied by an abrupt decrease in the weight of the sample in the TGA curve. These two are associated with the first stage of PAAm degradation, which includes an imidization reaction between the amino groups of the PAAm monomeric units, resulting into ammonia release.

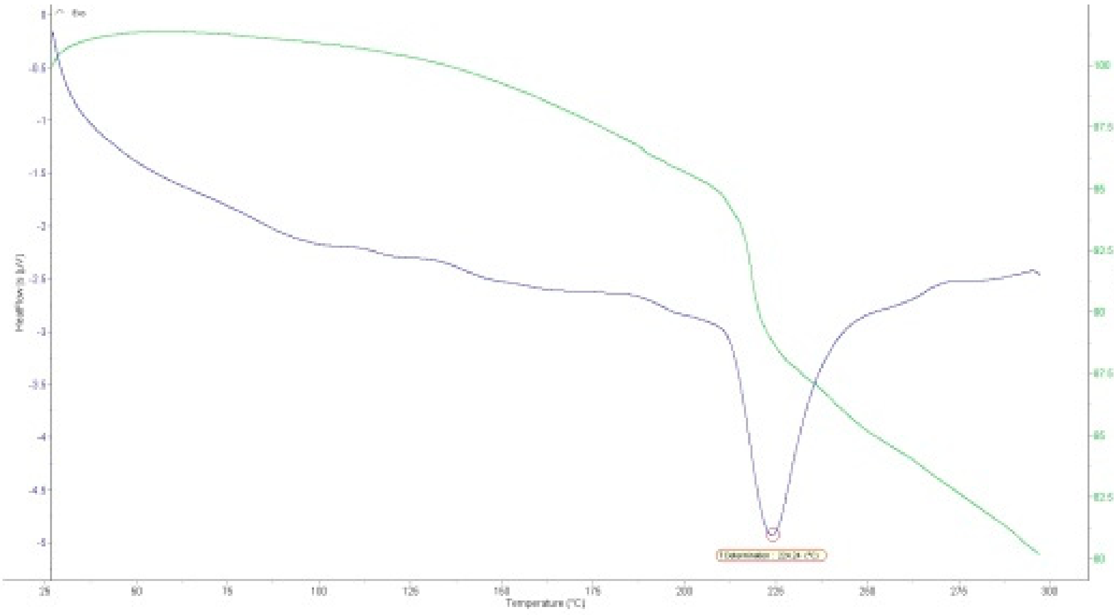

Figure S8. TGA (green line) and DTA (blue line) of PAAm single network.

There is a second stage of PAAm decomposition which occurs at 330-400°C and thus it is beyond the studied temperature region in our study, where the bonds in the polymer structure are broken as well as the imide bonds formed in the first stage, which stage is accompanied by CO_2 release.

In Figure S9 are presented the results obtained by TGA for the four IPNs. For comparison the TGA curves for both single networks (PAAm and PDMAEMA) are also provided. The four PDMAEMA/PAAm IPNs show a weight loss at ~ 220 °C, similarly to the single PAAm network, however for the IPNs this process is slower and takes place within a wider temperature range as compared to the single PAAm network. This is related to the "dilution" of the amino groups in the IPNs as compared to PAAm network due to the presence of the PDMAEMA chains. The amino groups drawing apart in the IPN structure results into slow-down of the PAAm degradation and reducing the weight loss of the IPNs due to the ammonia release during the first stage of PAAm degradation. Thus, the results obtained from DSC and TGA analysis show that PDMAEMA/PAAm IPNs are also a subject to thermal degradation, similar to the single PAAm network. While the temperature at which this degradation occurs is not significantly affected by the IPNs composition, the process is slower and takes place within a wider temperature region as compared to the single PAAm network.

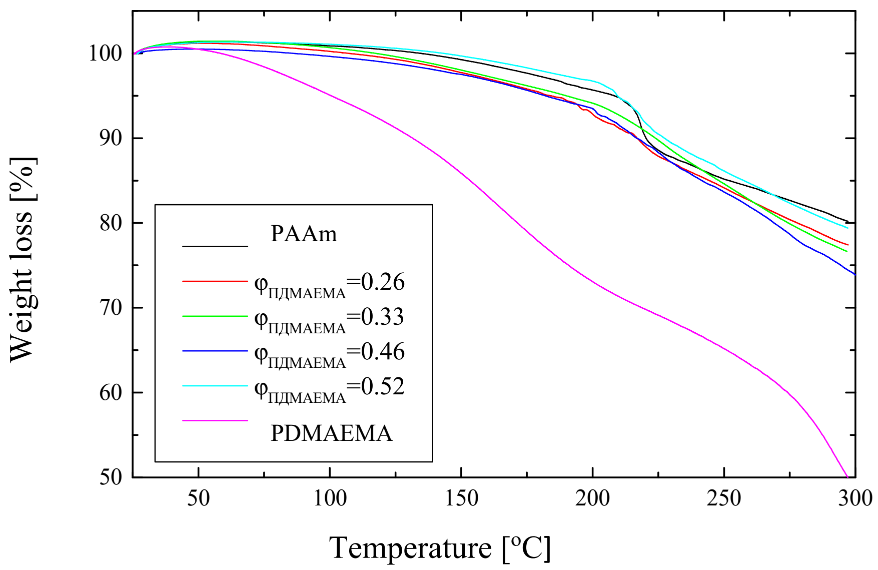

Figure S9. TGA of the four PAAm/PDMAEMA IPNs. The TGA curves for the single PAAm and PDMAEMA networks are also provided.

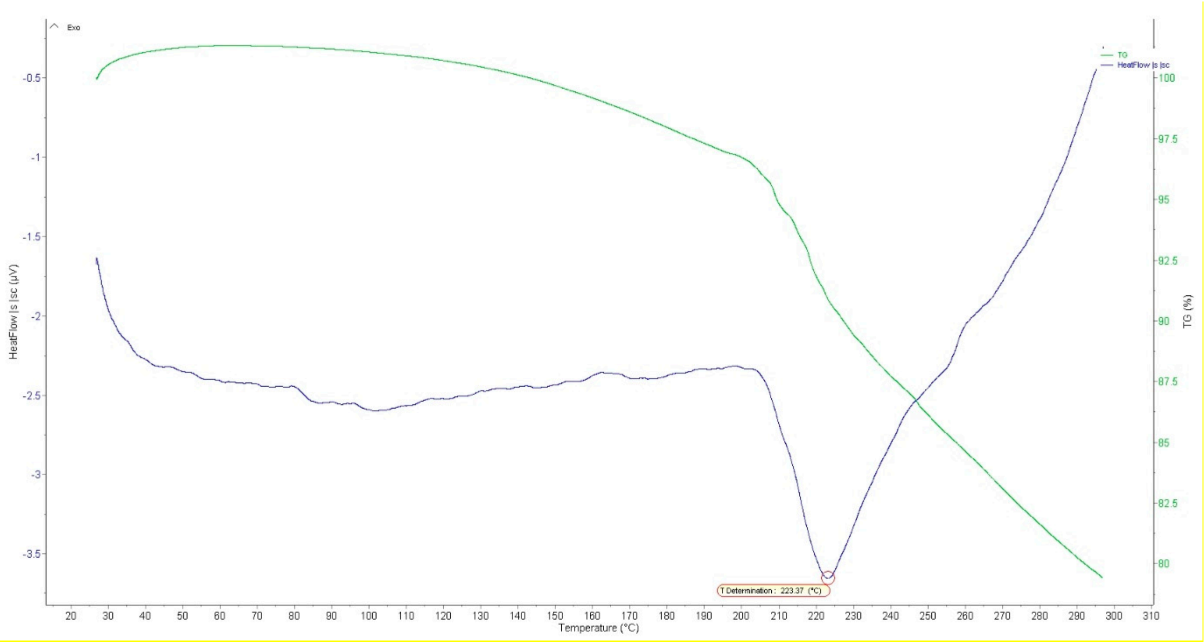

Figure S10. TGA (green line) and DTA (blue line) of IPN 4 ($\varphi_{PDMAEMA}$=0.52)

For multicomponent polymer systems (blends, copolymers, etc.) the T_g dependence on their composition is usually described by the Fox equation:

$$\frac{1}{T_{вст}} = \frac{\varphi_{ПДМАЕМА}}{T_{вст,ПДМАЕМА}} + \frac{\varphi_{ПААм}}{T_{вст,ПААм}} \qquad \text{(S5)}$$

where T_g^{PAAm} and $T_g^{PDMAEMA}$ are, respectively, the glass transition temperatures of both single PDMAEMA and PAAm networks.
